# 812 Burn Patient Tissue Fibroblasts Successfully Extracted to Study in Fibrotic Predictive Model

**DOI:** 10.1093/jbcr/iraf019.343

**Published:** 2025-04-01

**Authors:** Meredith Hanrahan, Jeffrey Anderson, Jennifer Patten, Karin Wang

**Affiliations:** Temple University Hospital; Temple University Hospital; Temple University; Temple University

## Abstract

**Introduction:**

After a burn, a tightly controlled wound repair process occurs. This often results in scar formation from a dysregulated replacement of extracellular matrix (ECM) and fibrosis, ultimately leading to loss of normal tissue function. Fibroblasts play a crucial role in wound healing by producing ECM components and facilitating tissue repair. Yet wound fibroblast dysregulation can directly provoke the fibrotic response. To develop a patient specific predictive model of fibrosis severity, we hypothesized collection of burned fibroblasts can be isolated from patient tissue samples.

**Methods:**

Human skin biopsy samples of 0.012” thickness were collected in the operating room during burn excisional debridement. Biopsied locations included full thickness burn, burn adjacent healthy tissue, and remote healthy tissue samples (donor site) depending on the clinical scenario. Samples were placed in tissue transport medium at 4° C and were transferred to the laboratory for processing. Biopsies, maintained in fibroblast medium, were divided into 2mm x 2mm portions. A single tissue portion was placed in the center of each well and fed with 500mL of fibroblast medium. Once fibroblast cells reached 75% confluency in a single well, the cells were detached from the tissue culture plate using Trypsin-EDTA solution and transferred to tissue culture flasks. Cells were collected from flasks and frozen for later study.

**Results:**

Biopsy samples were collected from three patients with full thickness burn injuries:

Patient 1: Three biopsies were taken five days post-injury: full-thickness burn, burn adjacent, and healthy skin. Time from tissue collection to sample plating was 172 minutes. Fibroblasts were collected from the full thickness burn sample on Day 13 and from healthy skin on Day 19.

Patient 2: One healthy skin biopsy was obtained 35 days post-injury. The time from tissue collection to plating was 65 minutes. Fibroblasts were collected on Day 30.

Patient 3: Two biopsies were taken 14 days post-injury: full thickness burn and burn adjacent tissue. The time from tissue collection to plating was 47 minutes. Fibroblasts were not collected due to insufficient migration out of tissue sample.

**Conclusions:**

Fibroblasts were obtained from healthy and full thickness burned samples, but not from burn adjacent tissue. This suggests there is a post-burn window, (here tested at Day 5 and Day 14), during which the fibroblasts in burn adjacent tissue significantly migrated into the wound to facilitate repair, depleting the level of fibroblasts in the nearby healthy tissue.

**Applicability of Research to Practice:**

We will further study these cells to determine how fibroblasts are regulated or dysregulated in normal and fibrotic wound healing within an engineered 2-D skin wound model. Here, we developed a patient-derived fibroblast collection protocol with the end goal of creating a model to predict fibrotic severity to inform clinical treatment.

**Funding for the Study:**

N/A

